# Association between the aMAP risk score and mortality in the MASLD/MetALD/ALD patient population: a cohort study

**DOI:** 10.3389/fmed.2026.1799986

**Published:** 2026-04-24

**Authors:** Peng-yang Li, Yuan-xin Mo, Rui-biao Fu, Jian Song, Shao-lei Kuang, Yuan-quan Zhao, Wei Jiang, Xiao-feng Dong

**Affiliations:** 1 Department of Hepatobiliary, Pancreas and Spleen Surgery, The People’s Hospital of Guangxi Zhuang Autonomous Region (Guangxi Academy of Medical Sciences), Nanning, China; 2 Department of Respiratory Oncology, Guangxi Medical University Cancer Hospital, Nanning, China; 3 Institute of Cardiovascular Sciences, Guangxi Academy of Medical Sciences and The People's Hospital of Guangxi Zhuang Autonomous Region, Nanning, China

**Keywords:** alcohol-related liver disease (ALD), metabolic and alcohol-related liver disease (MetALD), metabolic dysfunction-associated steatotic liver disease (MASLD), prognostic, the age–male–ALBI–platelets (aMAP) risk score

## Abstract

**Background:**

The age–male–ALBI–platelets (aMAP) risk score, an emerging non-invasive marker for liver fibrosis and hepatocellular carcinoma, has shown potential in risk stratification. However, its association with mortality in the broader population of Metabolic Dysfunction-Associated Steatotic Liver Disease (MASLD), MetALD, and Alcohol-related Liver Disease (ALD) remains unclear. Elucidating this relationship is crucial for healthcare and public health.

**Methods:**

We performed a cohort study using data from the National Health and Nutrition Examination Survey (NHANES) from 1999 to 2018. We used multivariable Cox proportional hazards models, Restricted cubic spline (RCS) analysis and Kaplan–Meier curves to assess the association between the aMAP score and all-cause, cardiovascular, and cancer mortality risks. The Fine-Grey competing risk analyses were used as a supplement. Mortality data were ascertained via the National Death Index through December 31, 2019. An independent hospital-based Southern Chinese cohort (*n* = 642) was additionally included for external validation of the association between aMAP score and MASLD.

**Results:**

A total of 32,654 participants were included. The prevalence of MASLD, MetALD, and ALD was 41.14, 2.22, and 0.79%, respectively. RCS analysis revealed a non-linear relationship between aMAP and all-cause mortality in all SLD subclassifications. Kaplan–Meier curves confirmed significantly lower survival rates in participants with higher aMAP scores. After multivariable adjustment, the high aMAP risk group (>60) had a significantly higher risk of all-cause, cardiovascular, and cancer mortality in most SLD classifications. This association remained robust in subgroup analyses for MASLD (HR: 1.11), MetALD (HR: 1.39), and ALD (HR: 1.87) on all-cause mortality. In the external validation cohort, elevated aMAP scores were also associated with higher odds of MASLD, showing an overall positive and approximately linear relationship. External validation demonstrated the linear association between aMAP and MASLD.

**Conclusion:**

The aMAP score is independently associated with long-term mortality risk across the whole subgroup of steatotic liver disease. As a readily available and effective risk-stratification tool, the aMAP stratification can help identify high-risk individuals within all SLD subclassifications and support clinical application and resource allocation. The association of aMAP with prevalence of MASLD was further supported by findings from an independent hospital-based validation cohort.

## 1 Introduction

Metabolic dysfunction-associated steatotic liver disease (MASLD), a new term replacing non-alcoholic fatty liver disease (NAFLD), has become the most common chronic liver disease worldwide, affecting about 30% of adults and presenting significant public health challenges (1). MASLD not only leads to severe hepatic complications such as cirrhosis and hepatocellular carcinoma, but is also closely associated with various extrahepatic manifestations, including cardiovascular disease and diabetes (2, 3). According to the 2023 multisociety Delphi consensus, steatotic liver disease (SLD) is primarily categorized into three distinct categories based on cardiometabolic risk factors (CMRFs) and alcohol consumption patterns: MASLD, metabolic and alcohol-related liver disease (MetALD), and alcohol-related liver disease (ALD) (4, 5). This refined classification system maintains continuity with existing clinical phenotypes while enhancing the accuracy of risk stratification and therapeutic relevance.

The oncogenic risk associated with SLD warrants particular attention, as it is a risk factor for both intrahepatic and extrahepatic malignancies. With the improved control of viral hepatitis, MASLD is increasingly recognized as a major cause of hepatocellular carcinoma (6). The characteristics and long-term outcomes of these newly defined subgroups, as well as the potential benefits of using noninvasive biomarkers within them, remain unclear. Recently, the age–male–ALBI–platelets (aMAP) risk score has shown excellent performance as a simple, objective, and accurate prognostic tool for assessing HCC risk in patients with various causes of hepatitis (7). Evidence also indicates that the aMAP score is a promising noninvasive tool for diagnosing fibrosis in CHB patients (8). Unlike traditional fibrosis scores such as Fibrosis-4(FIB-4), the aMAP score incorporates markers of hepatic synthesis and excretion, potentially offering a more comprehensive prognostic assessment. However, the utility of aMAP for assessing HCC risk and prognosis in the population with steatotic liver disease still requires validation using representative population-based data.

The National Health and Nutrition Examination Survey (NHANES) database, with its representative large-scale population-based data, provides an ideal resource for investigating liver disease. This study aims to comprehensively evaluate the associations between different SLD subclassifications and aMAP scores using NHANES data from 1999 to 2018, and to explore the predictive value of aMAP stratification for disease prognosis and mortality risk. These findings will enhance our understanding of the clinical characteristics under the new SLD definitions, optimize risk assessment strategies, and provide more robust guidance for clinical practice.

## 2 Methods

### 2.1 Data source

**Figure 1:**
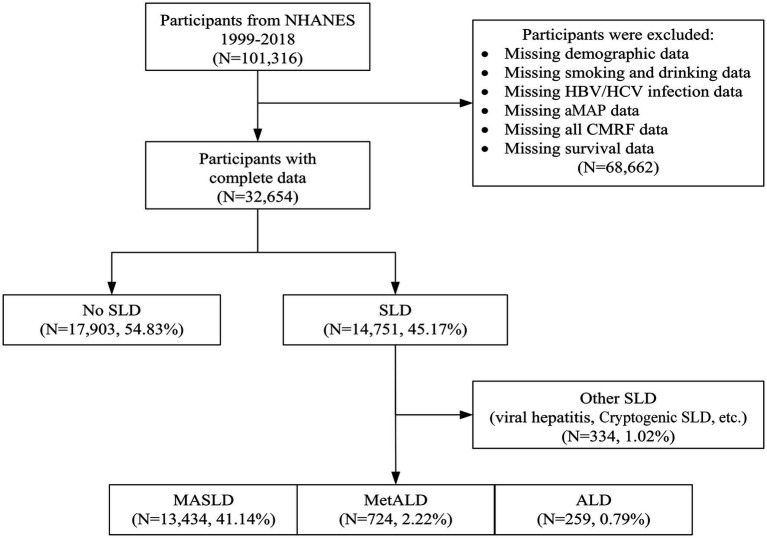
Flow diagram of the participant screening and enrollment process.

The NHANES database systematically collected nationally representative health-related data on the noninstitutionalized US population, using a stratified, multistage probability sampling design. NHANES was approved by the National Center for Health Statistics (NCHS) Ethics Review Board. See https://www.cdc.gov/nchs/nhanes/ for more information and a complete list of supported databases. A total of 101,316 participants aged 18 years and older were initially identified from the NHANES database covering ten consecutive cycles from 1999 to 2018. We sequentially excluded participants with missing demographics, covariates, and laboratory parameters essential for SLD subclassification and aMAP diagnosis. Finally, 32,654 participants were prospectively included in the subsequent analyses (Figure 1). In addition, multiple imputation was performed for missing covariates, including marital status, smoking status, and poverty income ratio (PIR), and 36,602 participants were ultimately included in the post-imputation sensitivity analysis.

For analyses combining NHANES 1999–2018 data, we used MEC examination weights because variables from the MEC component were included in this study. In accordance with NHANES analytic guidance for combining survey cycles, the 20-year MEC weight was constructed by rescaling the 4-year MEC examination weight for 1999–2002 as WTMEC4YR × 2/10 and the 2-year MEC examination weight for each cycle from 2003–2018 as WTMEC2YR × 1/10. To account for the complex survey design, we incorporated SDMVSTRA as the stratum variable and SDMVPSU as the primary sampling unit (PSU) variable.

### 2.2 Definition of steatotic liver disease subclassification

Steatotic liver disease is defined as hepatic steatosis with lipid accumulation exceeding 5% in hepatocytes. In this study, we assessed hepatic steatosis using the Fatty Liver Index (FLI), a widely validated noninvasive marker (9). A FLI ≥ 60 indicates the presence of hepatic steatosis. The FLI incorporates body mass index (BMI), waist circumference (WC), gamma-glutamyl transferase (GGT), and triglycerides (TG), and has shown good diagnostic performance in previous studies. In addition, we considered the US Fatty Liver Index (US-FLI), which was developed in the NHANES population, as a supplementary measure in exploratory analyses (10). A US-FLI ≥ 30 was regarded as indicating hepatic steatosis. All formulas are shown in Supplementary Table S1.

For the diagnosis of MASLD or MetALD, participants must meet at least one of the following five cardiometabolic risk factors (CMRFs) (4): (1) BMI ≥ 25 kg/m^2^ or WC ≥ 94 cm (males)/≥80 cm (females); (2) Dysglycemia: fasting glucose ≥5.6 mmol/L (100 mg/dL), 2-h post-load glucose ≥7.8 mmol/L (140 mg/dL), HbA1c ≥ 5.7% (39 mmol/L), diagnosis of type 2 diabetes, or current diabetes treatment; (3) Blood pressure ≥130/85 mmHg or antihypertensive medication; (4) Triglycerides ≥1.70 mmol/L (150 mg/dL) or lipid-lowering therapy; (5) HDL-cholesterol ≤1.0 mmol/L (40 mg/dL) in males or ≤1.3 mmol/L (50 mg/dL) in females, or lipid-lowering therapy.

Alcohol consumption was assessed using drinking questionnaires. One standard drink was defined as 12 oz. beer, 5 oz. wine, or 1.5 oz. liquor, each containing 14 grams of alcohol. Daily alcohol intake was calculated from 24-h dietary recalls using the USDA’s automated multiple-pass method. Based on the updated consensus guidelines, alcohol consumption was categorized in 3 groups as follows (4): (1) Light: <20 g/day (females) and <30 g/day (males); (2) Moderate: 20-50 g/day (females) and 30-60 g/day (males); (3) Excessive: >50 g/day (females) and >60 g/day (males). Excluding those individuals who had positive HBsAg and/or HCV RNA, the final diagnostic classifications were: (1) MASLD: SLD with ≥1 CMRF and light alcohol intake; (2) MetALD: SLD with ≥1 CMRF and moderate alcohol intake; (3) ALD: SLD with excessive alcohol intake regardless of CMRF.

### 2.3 Definition of noninvasive biomarkers

The aMAP risk score was calculated in eligible participants using the following formula (7): aMAP risk score = ((0.06 × age[year] + 0.89 × sex (Male: 1, Female: 0) + 0.48 × [(log₁₀ bilirubin[μmol/L] × 0.66) + (albumin[g/L] × −0.085)] – 0.01 × platelets[10^9^/L]) + 7.4)/14.77 × 100. The aMAP score <50, 50–60, and >60 was categorized as low, medium, and high risk group, respectively.

The FIB-4 score was calculated using the formula (11): FIB-4 score = (Age [year] × AST [U/L])/((platelets [10^9^/L]) × (ALT [U/L])^(1/2)^). We stratified those with participants into three risk categories: FIB-4 < 1.3, 1.3–2.67, and >2.67, respectively. Albumin-Bilirubin (ALBI) (12), Metabolic Dysfunction-Associated Fibrosis 5 (MAF-5) (13), and NAFLD fibrosis score (NFS) (14) are frequently used to evaluate prognosis. These formulas are also set in Supplementary Table S1.

### 2.4 Assessment of covariates

We included comprehensive demographic characteristics, including sex (male, female), age, race/ethnicity (non-Hispanic White/non-Hispanic Black/Mexican American/Other race), marital status (married/previously married/never married), educational level (less than high school/high school/more than college), and poverty-income ratio (PIR, with a higher ratio representing a higher level of income). Additionally, we collected data on lifestyle factors and comorbidities, including smoking status and histories of diabetes and hypertension. Besides essential physical examinations and laboratory tests, we also documented liver enzymes, including alanine aminotransferase (ALT) and aspartate aminotransferase (AST). These covariates were incorporated into analyses to evaluate whether the aMAP score varied across these potential confounding factors.

### 2.5 All-cause and cause-specific mortality

All-cause and cause-specific mortality were determined through linkage with the National Death Index (NDI), with follow-up through December 31, 2019^1^. In this study, the primary outcome was all-cause mortality, and follow-up time was defined as the interval from the date of the NHANES baseline examination to the date of death or the end of follow-up, whichever came first. Other cause-specific mortality, with underlying causes of death identified using the International Classification of Diseases (ICD) coding system. In our study, we mainly examined cardiovascular and cancer mortality. While comprehensive mortality data were available for most major causes, the dataset did not include liver disease-specific mortality. Mortality patterns were analyzed across SLD subclassifications to assess prognostic differences.

### 2.6 External validation cohort

To evaluate the external applicability of the primary findings, an independent hospital-based cohort from the People’s Hospital of Guangxi Zhuang Autonomous Region was established. A total of 642 consecutive participants were included, all of whom had complete abdominal ultrasonography data and available laboratory measurements required for calculation of the aMAP score. Hepatic steatosis was assessed using standardized abdominal ultrasonography performed by experienced radiologists according to routine clinical practice. Serum albumin, total bilirubin, and platelet counts were obtained from fasting blood samples collected during the same visit, and the aMAP score was calculated using the established formula. We included only the data required for MASLD diagnosis and aMAP calculation. This external cohort was used to independently assess the cross-sectional relationship between aMAP score and prevalent MASLD observed in the population-based analysis. The variables included in the external analysis were limited to MASLD status and aMAP score. The study protocol was reviewed and approved by the Ethics Committee of the People’s Hospital of Guangxi Zhuang Autonomous Region (Approval No. KY-IIT-2025-83), and the study was conducted in accordance with the principles of the Declaration of Helsinki.

### 2.7 Statistical analysis

Data analysis in this study was conducted in accordance with CDC statistical analysis guidelines. We described baseline characteristics according to SLD subclassifications. Comparisons of characteristics between SLD groups were performed using the Kruskal-Wallis test or Wilcoxon rank-sum test for continuous variables, and chi-square test or Fisher’s exact test for categorical variables as appropriate. Multiple imputation was performed to assess the robustness of the analytical dataset. NHANES data from 1999 to 2018 were divided into 10 survey cycles to describe temporal trends in the distribution of aMAP risk categories across different SLD subgroups. Time-dependent area under the curve (AUC), sensitivity, specificity, positive predictive value (PPV), and negative predictive value (NPV) were used to evaluate the predictive ability of aMAP, ALBI, FIB-4, MAF-5, and NFS for mortality. The Delong approach was employed to ascertain whether there were statistically significant disparities in AUC between noninvasive scores. Three models were constructed: Model 1 was unadjusted; Model 2 was adjusted for race/ethnicity; Model 3 additionally included socioeconomic variables such as PIR, education level, marital status and smoking. Subgroup analyses were conducted to assess all-cause mortality across SLD subclassifications with different model adjustments. Restricted cubic spline (RCS) analyses were performed to examine the potential nonlinear associations of aMAP scores with MASLD prevalence and all-cause mortality across SLD subclassifications. Three knots were placed at the 25th, 50th, and 75th percentiles of the aMAP distribution. The RCS curves were used to visualize the dose–response relationships and to explore potential threshold effects. Multivariable Cox proportional hazards models were employed to identify key variables influencing all-cause mortality, including SLD subclassifications, age, sex, race/ethnicity, education, marital status, PIR, smoking, and aMAP categories. To assess the appropriateness and robustness of the Cox regression models, proportional hazards assumption testing and a 2-year landmark analysis were additionally performed (Supplementary Tables S2, S3). Kaplan–Meier survival analysis was performed to compare survival outcomes among different aMAP score categories in participants with MASLD, MetALD, and ALD. The Fine-Grey competing risk analyses were used to determine the cumulative incidence of cause-specific mortality in each SLD subclassification and aMAP score category, respectively. Subgroup analyses further examined the associations of the aMAP high-risk group (aMAP> 60) versus other groups (aMAP≤ 60) with all-cause mortality, cardiovascular mortality, and cancer mortality across different SLD subclassifications. For the independent external validation cohort, the association between aMAP score and MASLD was examined using logistic regression models. Restricted cubic spline analyses were conducted to explore the dose–response relationship between aMAP score and MASLD and to assess potential nonlinearity. All statistical analyses were performed using R software (version 4.3.2; R Foundation for Statistical Computing, Vienna, Austria). Two-sided *p* values < 0.05 were considered statistically significant.

## 3 Results

### 3.1 Baseline characteristics

A total of 101,316 participants were enrolled in the NHANES 1999–2018 dataset. After excluding 68,662 participants with missing data on demographics, smoking status, alcohol consumption, HBV/HCV infection, aMAP score, CMRFs, and survival outcomes, 32,654 participants were included in the primary analysis (Figure 1). Among these, 14,751 (45.17%) had hepatic steatosis, comprising 13,434 (41.14%) with MASLD, 724 (2.22%) with MetALD, and 259 (0.79%) with ALD. The remaining 334 participants (1.02%) with other forms of SLD were excluded from further analysis.

The baseline characteristics according to SLD classification are summarized in Table 1. Compared to participants without SLD, those with SLD were older, more likely to be male and Mexican American, with significantly higher prevalence of hypertension (65.8–73.0%) and diabetes (17.0–33.0%). The distribution of CMRFs differed markedly: while 82% of participants without SLD still harbored at least one CMRF, those with MASLD predominantly had ≥3 CMRFs (45.3%), whereas MetALD and ALD patients more commonly presented with 2 CMRFs (34.0 and 32.4%, respectively).

**Table 1:** Baseline characteristics of NHANES 1999–2018 participants.

Variable	No SLD *N* = 17,903	MASLD *N* = 13,434	MetALD *N* = 724	ALD *N* = 259	*P-*value
Age, Mean (SD)	45.6 ± 18.7	51.0 ± 16.9	47.9 ± 15.2	47.2 ± 14.0	<0.001
Sex, *n* (%)					<0.001
Male	7,904 (44.1)	7,083 (52.7)	521 (72.0)	219 (84.6)	
Female	9,999 (55.9)	6,351 (47.3)	203 (28.0)	40 (15.4)	
Race, *n* (%)					<0.001
Mexican American	2,720 (15.2)	2,823 (21.0)	138 (19.1)	57 (22.0)	
Other Hispanic	3,204 (17.9)	1,861 (13.9)	68 (9.4)	36 (13.9)	
Non-Hispanic White	8,844 (49.4)	6,367 (47.4)	414 (57.2)	115 (44.4)	
Non-Hispanic Black	3,135 (17.5)	2,383 (17.7)	104 (14.4)	51 (19.7)	
Marita, *n* (%)					<0.001
Married	9,203 (51.4)	7,811 (58.1)	355 (49.0)	93 (35.9)	
Widowed/Divorced/Separated	3,391 (18.9)	2,980 (22.2)	169 (23.3)	73 (28.2)	
Never married	5,309 (29.7)	2,643 (19.7)	200 (27.6)	93 (35.9)	
Education, *n* (%)					<0.001
Below high school	3,981 (22.2)	3,637 (27.1)	171 (23.6)	97 (37.5)	
High school	3,897 (21.8)	3,233 (24.1)	205 (28.3)	81 (31.3)	
Above high school	10,025 (56.0)	6,564 (48.9)	348 (48.1)	81 (31.3)	
PIR, Mean (SD)	2.7 ± 1.6	2.5 ± 1.6	2.8 ± 1.7	2.1 ± 1.5	<0.001
Smoking, n (%)	10,238 (57.2)	7,090 (52.8)	196 (27.1)	55 (21.2)	<0.001
Drinking, Mean (SD), g/week					<0.001
Male	68.3 ± 136.0	33.5 ± 50.7	287.2 ± 56.6	704.2 ± 467.4	
Female	26.0 ± 66.9	9.0 ± 21.3	200.5 ± 56.8	678.0 ± 637.1	
CMRFs, *n* (%)					<0.001
0	3,229 (18.0)	0 (0.0)	0 (0.0)	5 (1.9)	
1	5,868 (32.8)	1,385 (10.3)	83 (11.5)	29 (11.2)	
2	4,590 (25.6)	3,341 (24.9)	246 (34.0)	84 (32.4)	
3	2,153 (12.0)	3,417 (25.4)	179 (24.7)	72 (27.8)	
4	1,234 (6.9)	2,622 (19.5)	120 (16.6)	42 (16.2)	
5	829 (4.6)	2,669 (19.9)	96 (13.3)	27 (10.4)	
Hypertension, *n* (%)	7,180 (40.1)	8,840 (65.8)	515 (71.1)	189 (73.0)	<0.001
Diabetes, *n* (%)	1,818 (10.2)	4,439 (33.0)	148 (20.4)	44 (17.0)	<0.001

SLD: steatotic liver disease; MASLD: Metabolic dysfunction-associated steatotic liver disease; MetALD: metabolic and alcohol-related liver disease; ALD: alcohol-related liver disease; SD: standard deviation; CMRFs: cardiometabolic risk factors. Data presented as mean (SD) or n (%). Kruskal-Wallis test or Wilcoxon rank-sum test for continuous variables, and chi-square test or Fisher’s exact test for categorical variables.

As shown in Table 2, liver enzymes and metabolic parameters were significantly elevated across all SLD classifications. The distribution of aMAP scores revealed significant prognostic stratification: 40% of MASLD patients and 45% of both MetALD and ALD participants were classified as medium-to-high risk (aMAP ≥50), compared to only 29% in the no SLD group. Notably, approximately 10% of participants across all SLD subtypes were categorized as aMAP high-risk (>60). Additionally, the proportion of participants with FIB-4 > 2.67 increased progressively from MASLD (3.1%) to MetALD (5.5%) and ALD (8.5%), respectively (*p* < 0.001). The weighted baseline characteristics and prevalence estimates are now presented in Supplementary Tables S4, S5. These weighted results demonstrate patterns that are generally consistent with our original unweighted analyses. The baseline characteristics after multiple imputation are presented in Supplementary Table S6. No significant differences were observed before and after imputation in the distributions of SLD classifications, age, sex, aMAP score, or mortality (all *p* > 0.05).

**Table 2:** Laboratory characteristics of NHANES 1999–2018 participants.

Variable	No SLD *N* = 17,903	MASLD *N* = 13,434	MetALD *N* = 724	ALD *N* = 259	*p*-value
AST, U/L	22 (19, 26)	23 (19, 28)	26 (22, 34)	30 (24, 40)	<0.001
ALT, U/L	18 (15, 24)	24 (18, 33)	28 (21, 41)	34 (23, 49)	<0.001
Platelets, 10^9^/L	243 (206, 285)	249 (209, 295)	243 (204, 286)	234 (198, 274)	<0.001
Triglycerides, mg/dL	91 (66, 129)	163 (114, 241)	170 (118, 256)	174 (118, 273)	<0.001
Total Bilirubin, μmol/L	12.0 (8.6, 13.7)	10.3 (8.6, 13.7)	12.0 (8.6, 15.4)	12.0 (8.6, 15.4)	<0.001
Albumin, g/L	43 (41, 45)	42 (40, 44)	43 (41, 45)	43 (41, 45)	<0.001
aMAP score	43 (37, 52)	47 (40, 55)	48 (41, 54)	48 (42, 56)	<0.001
aMAP group, *n* (%)					<0.001
<50	12,650 (71%)	8,029 (60%)	441 (61%)	143 (55%)	
50–60	3,681 (21%)	3,909 (29%)	210 (29%)	90 (35%)	
≥60	1,572 (8.8%)	1,496 (11%)	73 (10%)	26 (10%)	
FIB-4 score	0.85 (0.57, 1.33)	0.94 (0.62, 1.39)	0.94 (0.63, 1.47)	1.02 (0.67, 1.63)	<0.001
FIB-4 group, *n* (%)					<0.001
<1.3	13,221 (74%)	9,556 (71%)	498 (69%)	163 (63%)	
1.3–2.67	4,138 (23%)	3,463 (26%)	186 (26%)	74 (29%)	
> 2.67	544 (3.0%)	415 (3.1%)	40 (5.5%)	22 (8.5%)	
MAF-5 score	−1.41 (−2.23, −0.47)	1.21 (0.00, 2.78)	1.08 (−0.07, 2.63)	1.37 (0.32, 2.98)	<0.001
NFS score	−2.57(−3.40, −1.55)	−1.36(−2.44, −0.21)	−1.89(−2.82, −0.84)	−1.85(−2.73, −0.69)	<0.001
ALBI score	−2.69(−3.16, −2.78)	−2.90(−3.10, −2.74)	−2.99(−3.16, −2.77)	−2.95(−3.16, −2.77)	<0.001

SLD: steatotic liver disease; MASLD: Metabolic dysfunction-associated steatotic liver disease; MetALD: metabolic and alcohol-related liver disease; ALD: alcohol-related liver disease; ALT: alanine aminotransferase; AST: aspartate transaminase; ALBI score: albumin-bilirubin score; aMAP: age–male–ALBI–platelets; FIB-4: Fibrosis-4; MAF-5: metabolic dysfunction–associated fibrosis-5; NFS: NAFLD fibrosis score. Data presented as median (Q1, Q3) or n (%). Kruskal-Wallis test or Wilcoxon rank-sum test for continuous variables, and chi-square test or Fisher’s exact test for categorical variables.

**Figure 2:**
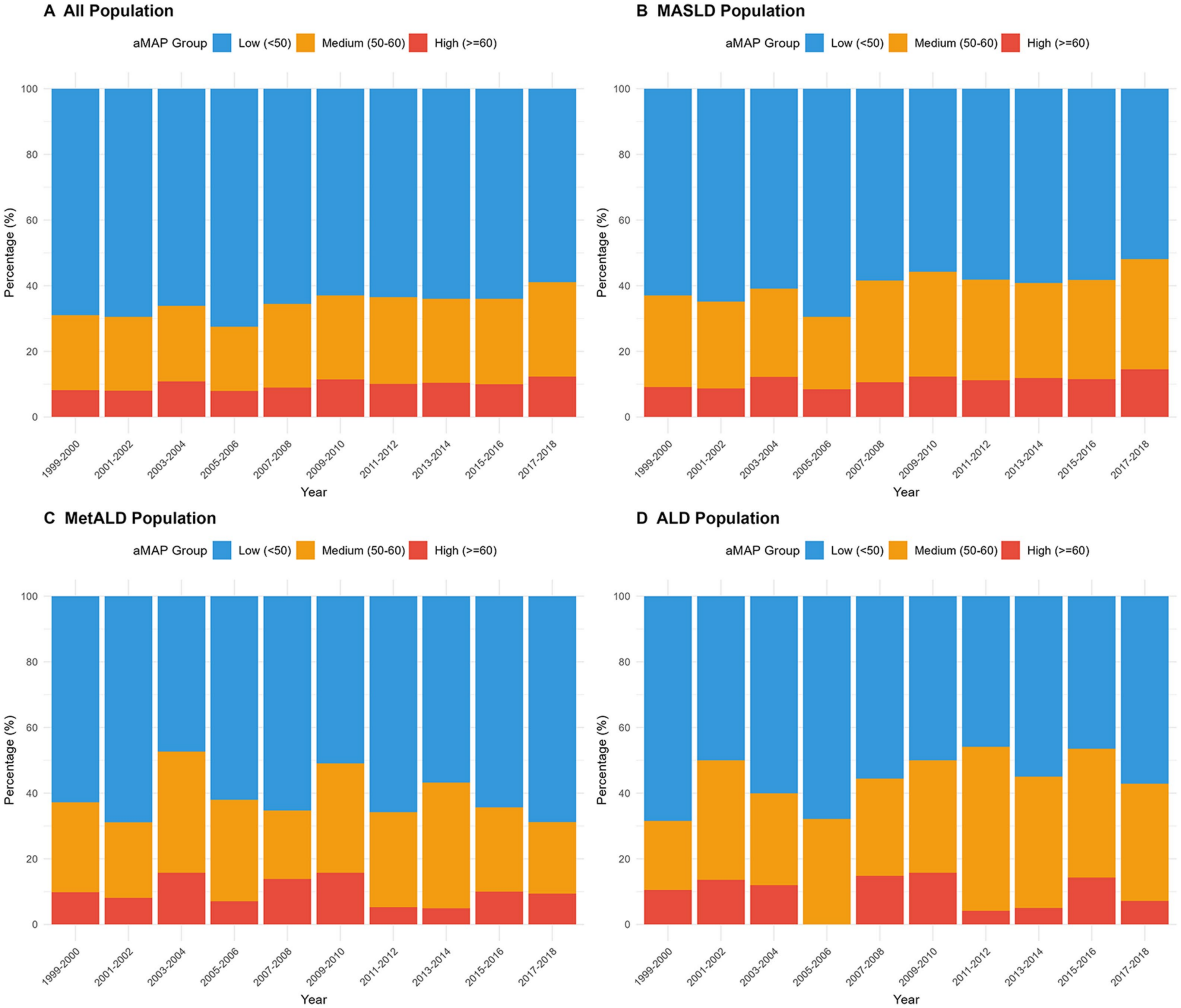
Distribution of aMAP risk categories across NHANES 1999–2018 cycles by population subgroup. The proportion of low (<50), medium (50–60), and high (>60) aMAP risk categories across 10 NHANES cycles in all population **(A)**, MASLD **(B)**, MetALD **(C)**, and ALD **(D)**, respectively. SLD: Steatotic liver disease; MASLD: Metabolic dysfunction-associated steatotic liver disease; MetALD: Metabolic and alcohol-related liver disease; ALD: Alcohol-related liver disease; aMAP: Age–male–ALBI–platelets.

Before subsequent analyses, we employed weighted analysis. We examined the distribution of aMAP risk categories across 10 NHANES cycles (1999–2018) by population subgroup (Figures 2A–D). The proportion of medium- to high-risk participants increased over time in the overall population and MASLD subgroup, exceeding 40% in some cycles. MetALD and ALD populations showed irregular distributions due to smaller sample sizes.

**Figure 3:**
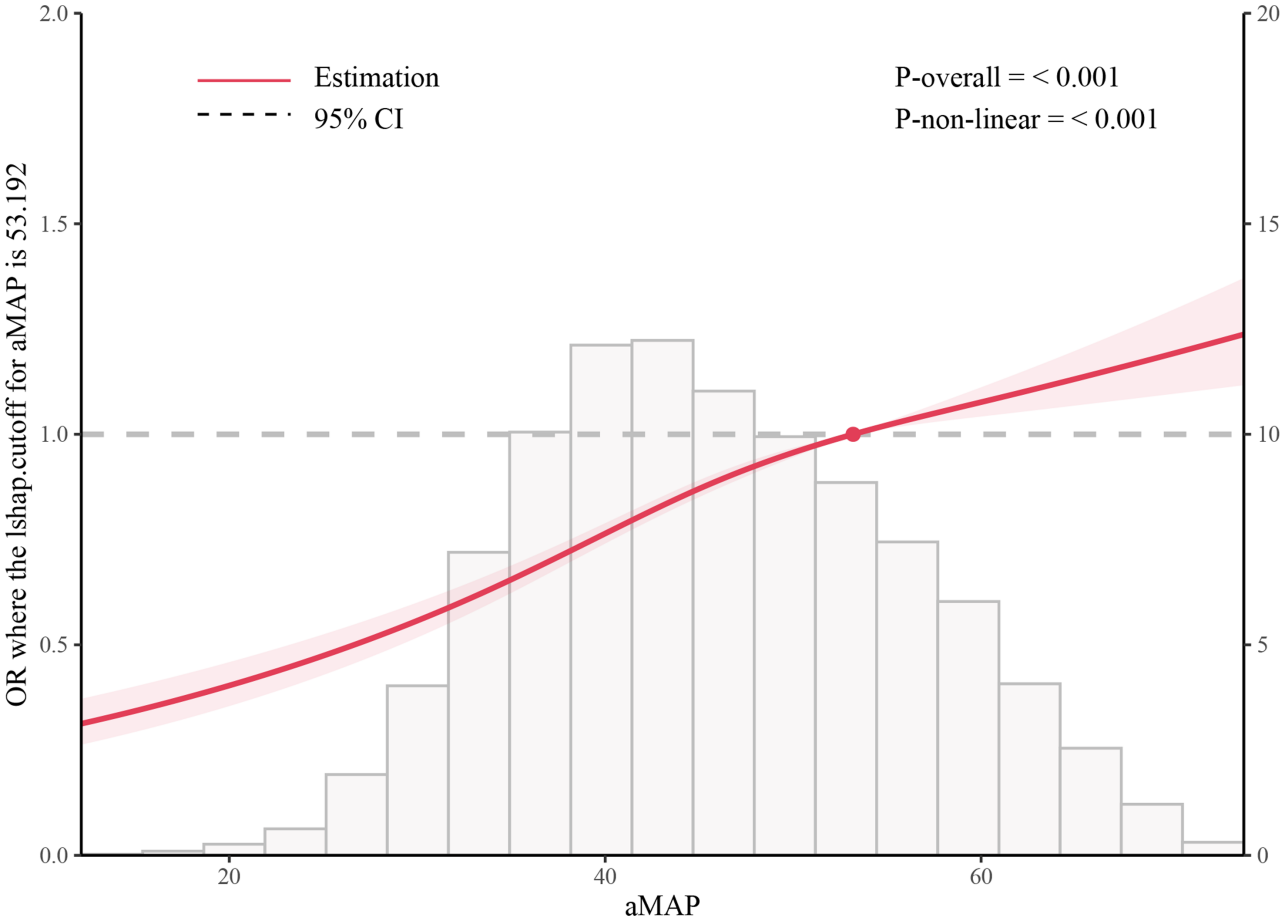
Restricted cubic spline analysis for the association between aMAP score and MASLD in the NHANES cohort. The solid line represents the estimated odds ratio (OR), and the shaded area indicates the 95% confidence interval. The reference value was set at an aMAP score of 53.192. *P*-overall < 0.001 and *P*-non-linear < 0.001.

In addition, RCS analysis based on the NHANES cohort demonstrated a significant association between aMAP score and the presence of MASLD (*P*-overall < 0.001), with clear evidence of a non-linear dose–response relationship (*P*-non-linear < 0.001). Using an aMAP value of 53.192 as the reference point, the odds of MASLD increased progressively with higher aMAP scores (Figure 3).

### 3.2 Survival of individuals with SLD and prediction by the aMAP score

**Figure 4:**
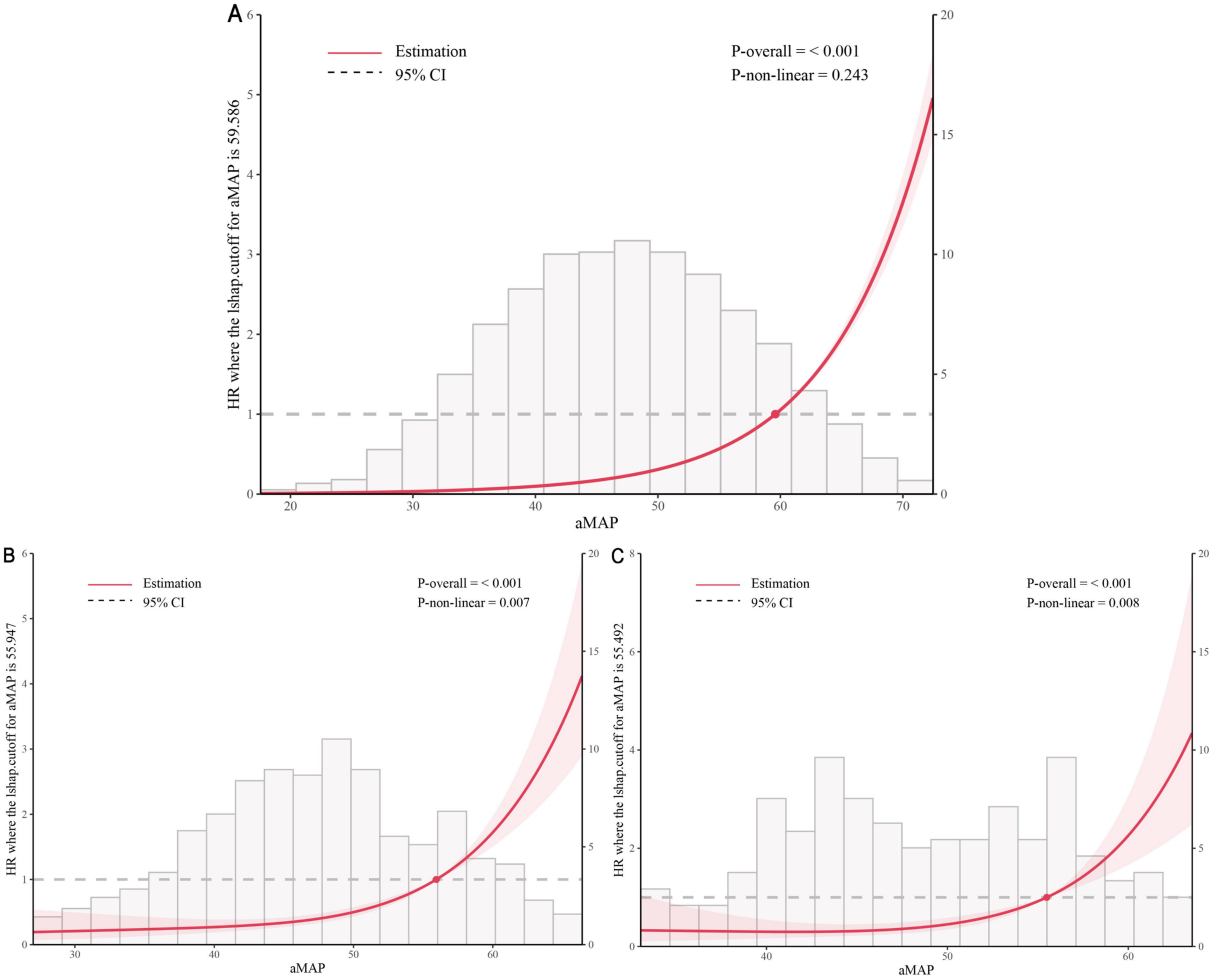
Restricted cubic splines reflect the relationships between aMAP score and all-cause mortality among adults with SLD subclassifications, including MASLD **(A)**, MetALD **(B)**, and ALD **(C)**, respectively. The model was adjusted for race/ethnicity, poverty income ratio, education level, marital status, and smoking.

With a maximum follow-up of 20 years in the NHANES 1999–2018 cohort, we evaluated the association between aMAP score and all-cause mortality across SLD classifications. Before the analysis, we constructed three models by adjusting for covariates through multivariate Cox regression analysis (Supplementary Table S7). The comparative AUCs of aMAP and its individual components are shown in Supplementary Table S8. Age alone yielded AUC values of 0.819, 0.837, and 0.872 for predicting 5-, 10-, and 20-year all-cause mortality, respectively, which were slightly higher than those of aMAP (*p* < 0.05). In Table 3, we compared five indices: aMAP, ALBI, FIB-4, MAF-5, and NFS for their ability to predict all-cause mortality at 5, 10, and 20 years of follow-up in the SLD population. aMAP consistently exhibited the highest AUC across all time points (5-year: 0.788; 10-year: 0.809; 20-year: 0.833), outperforming other non-invasive indexes. DeLong’s test demonstrated that the AUC of aMAP was significantly higher than other non-invasive indices (all *p* < 0.05). While PPV and NPV varied among scores, aMAP demonstrated superior overall discriminative association for long-term mortality. To minimize the potential influence of age on the prognostic performance of the aMAP score, we further compared the time-dependent ROC performance of aMAP with other composite indices in subgroups stratified by an age cutoff of 60 years in Supplementary Table S9. The results showed that aMAP maintained good prognostic discrimination after age stratification and remained among the best-performing composite indices in both age groups. After adjusting for sociodemographic factors (race, education, smoking, poverty income ratio, and marital status), RCS analysis revealed significant relationships between aMAP score and mortality in all three SLD subclassifications (Figures 4A–C), and L-shaped curves were observed for MASLD, MetALD, and ALD (*p* < 0.001), with all-cause mortality risk in SLD populations showing a consistent upward trend beyond aMAP score thresholds of approximately 60, 56, and 55, respectively. As shown in Supplementary Figure S1, the RCS analyses based on the multiply imputed dataset demonstrated trends in all-cause mortality and disease burden across aMAP and SLD subclassifications that were consistent with those observed before imputation.

**Table 3:** Predictive performance of noninvasive biomarkers for all-cause mortality among the total SLD population.

Variable name	AUC	Threshold	Sensitivity	Specificity	PPV	NPV
5 years						
aMAP	0.79 (0.77–0.81)	41.95	0.79 (0.77–0.81)	0.93 (0.91–0.94)	0.32 (0.31–0.33)	0.99 (0.98–0.99)
FIB-4	0.77 (0.76–0.79)	0.79	0.77 (0.76–0.79)	0.90 (0.88–0.92)	0.41 (0.40–0.41)	0.99 (0.98–0.99)
MAF-5	0.63 (0.61–0.65)	0.97	0.63 (0.61–0.65)	0.74 (0.70–0.77)	0.47 (0.46–0.47)	0.97 (0.97–0.97)
ALBI	0.60 (0.58–0.62)	−2.81	0.60 (0.58–0.62)	0.51 (0.48–0.55)	0.64 (0.63–0.65)	0.96 (0.96–0.96)
NFS	0.76 (0.75–0.78)	−2.28	0.76 (0.75–0.78)	0.91 (0.89–0.93)	0.30 (0.29–0.31)	0.98 (0.98–0.99)
10 years						
aMAP	0.81 (0.80–0.82)	42.91	0.81 (0.80–0.82)	0.92 (0.91–0.93)	0.37 (0.37–0.38)	0.98 (0.97–0.98)
FIB-4	0.80 (0.79–0.81)	0.75	0.80 (0.79–0.81)	0.92 (0.90–0.93)	0.39 (0.38–0.40)	0.98 (0.97–0.98)
MAF-5	0.66 (0.64–0.67)	1.05	0.66 (0.64–0.67)	0.71 (0.68–0.73)	0.49 (0.48–0.50)	0.93 (0.93–0.94)
ALBI	0.58 (0.57–0.60)	−2.70	0.58 (0.57–0.60)	0.37 (0.35–0.40)	0.76 (0.75–0.77)	0.91 (0.90–0.92)
NFS	0.79 (0.77–0.80)	−2.13	0.79 (0.77–0.80)	0.90 (0.89–0.92)	0.35 (0.35–0.36)	0.97 (0.96–0.97)
20 years						
aMAP	0.83 (0.81–0.86)	45.61	0.83 (0.81–0.86)	0.86 (0.85–0.88)	0.49 (0.49–0.50)	0.95 (0.95–0.96)
FIB-4	0.82 (0.79–0.84)	0.96	0.82 (0.79–0.84)	0.80 (0.79–0.82)	0.58 (0.57–0.59)	0.94 (0.94–0.95)
MAF-5	0.67 (0.64–0.71)	1.98	0.67 (0.64–0.71)	0.48 (0.46–0.50)	0.66 (0.65–0.67)	0.87 (0.87–0.88)
ALBI	0.71 (0.67–0.74)	−2.59	0.71 (0.67–0.74)	0.19 (0.17–0.20)	0.87 (0.86–0.88)	0.85 (0.85–0.86)
NFS	0.81 (0.78–0.84)	−1.23	0.81 (0.78–0.84)	0.74 (0.72–0.75)	0.59 (0.59–0.60)	0.92 (0.92–0.93)

aMAP risk score: age–male–ALBI–platelets risk score; FIB-4 score: fibrosis-4; ALBI score: albumin-bilirubin score; MAF-5: metabolic dysfunction–associated fibrosis-5; NFS: NAFLD fibrosis score; AUC: area under the curve; PPV: Positive predictive value; NPV: Negative predictive value. DeLong’s test for aMAP compared with other non-invasive indices.

**Figure 5:**
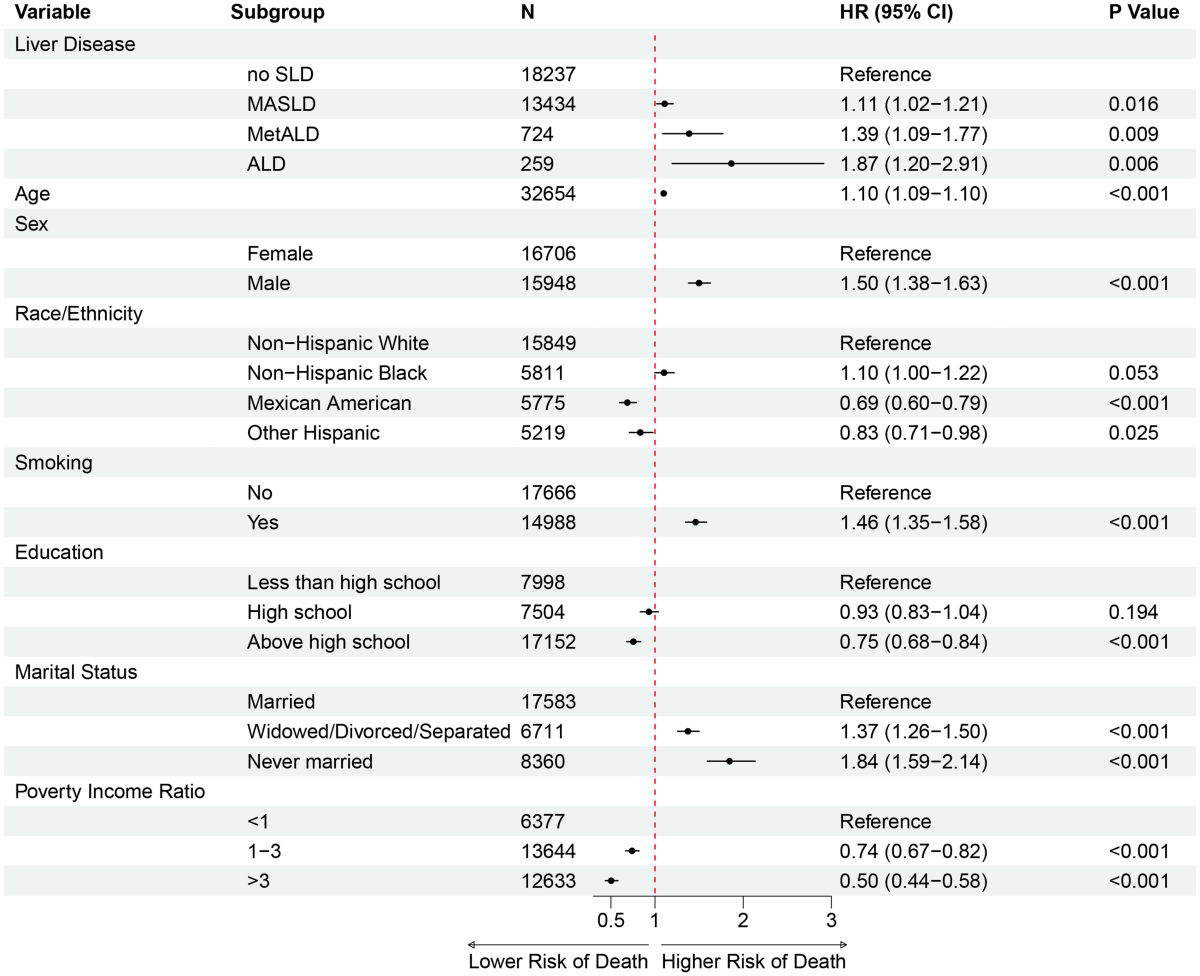
Forest plots showing multivariable Cox proportional hazard regressions for all-cause mortality of participants in each group. Including SLD subclassifications, age, sex, race/ethnicity, education, marital status, PIR, and smoking. MASLD: Metabolic dysfunction-associated steatotic liver disease; MetALD: metabolic and alcohol-related liver disease; ALD: alcohol-related liver disease; HR: hazard ratio; CI, confidence interval.

Multivariable Cox regression analysis incorporating SLD subclassifications and sociodemographic factors is presented in Figure 5. Compared to no SLD, MASLD, MetALD and ALD were associated with 11% (HR 1.11, 95% CI: 1.02–1.21), 39% (HR 1.39, 95% CI: 1.09–1.77), and 87% (HR 1.87, 95% CI: 1.20–2.91) higher mortality risk, respectively. Other significant predictors included age (HR 1.10 per year), Male (HR 1.50, 95% CI: 1.38–1.63), Mexican American ethnicity (HR 0.69, 95% CI: 0.60–0.79), current smoking (HR 1.46, 95% CI: 1.35–1.58), and socioeconomic factors, including education, marital status and poverty income ratio.

Table 4 presents the risk assessment of all-cause and cause-specific mortality among participants with MASLD, MetALD, and ALD stratified by aMAP score into two groups. Compared to individuals without SLD, all liver disease subgroups demonstrated elevated all-cause mortality risk, except MASLD patients with aMAP ≤ 60. Cardiovascular mortality was predominantly increased among MASLD patients. Notably, when aMAP > 60, all SLD subclassifications exhibited varying degrees of elevated cancer mortality. Additionally, we conducted competing risk analyses to validate these findings, with the cumulative incidence of death from cardiovascular and cancer causes in Supplementary Tables S10, S11.

**Table 4:** Effect of SLD subclassifications on all-cause and cause-specific mortality stratified by aMAP score.

Subgroup	All-cause mortality				Cardiovascular mortality			Cancer mortality		
	*N*	Deaths	HR (95% CI)	*p*-value	Deaths	HR (95% CI)	*p*-value	Deaths	HR (95% CI)	*p*-value
No SLD	17,903	2,247	Reference		675	Reference		507	Reference	
MASLD (aMAP ≤ 60)	11,938	1,382	1.01 (0.91–1.11)	0.892	441	1.11 (0.96–1.29)	0.172	321	1.01 (0.84–1.22)	0.897
MASLD (aMAP > 60)	1,496	700	5.91 (5.18–6.74)	<0.001	268	7.48 (6.00–9.32)	<0.001	144	6.03 (4.76–7.64)	<0.001
MetALD (aMAP ≤ 60)	651	83	1.16 (0.87–1.55)	0.302	24	0.98 (0.56–1.70)	0.937	18	0.93 (0.53–1.64)	0.797
MetALD (aMAP > 60)	73	35	4.92 (3.21–7.54)	<0.001	9	6.29 (3.12–12.69)	<0.001	11	5.88 (2.85–12.14)	<0.001
ALD (aMAP ≤ 60)	233	32	1.16 (0.69–1.95)	0.567	9	0.65 (0.30–1.43)	0.289	7	1.87 (0.69–5.05)	0.215
ALD (aMAP > 60)	26	15	8.39 (4.16–16.94)	<0.001	4	8.20 (2.94–22.89)	<0.001	4	18.52 (4.43–77.49)	<0.001

Adjusted for race, education, smoking, poverty income ratio, and marital status. SLD: steatotic liver disease; MASLD: Metabolic dysfunction-associated steatotic liver disease; MetALD: metabolic and alcohol-related liver disease; ALD: alcohol-related liver disease; HR: hazard ratio; CI, confidence interval; aMAP: the age–male–ALBI–platelets.

**Figure 6:**
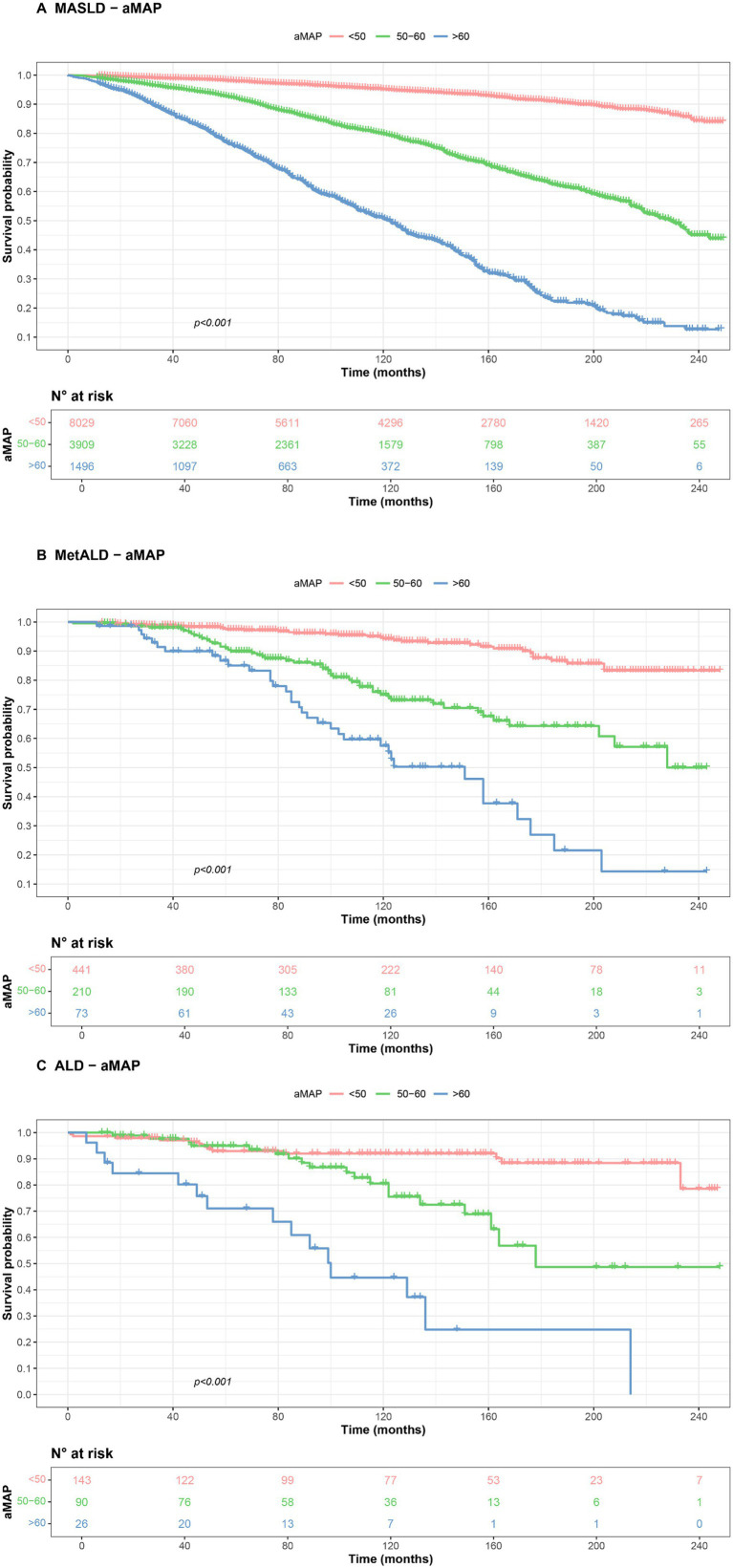
Kaplan–Meier survival curve for all-cause mortality among participants with MASLD/MetALD/ALD, including MASLD **(A)**, MetALD **(B)**, and ALD **(C)**, respectively. aMAP: Age–Male–ALBI–Platelets. MASLD: Metabolic dysfunction-associated steatotic liver disease; MetALD: metabolic and alcohol-related liver disease; ALD: alcohol-related liver disease.

In Figure 6, we further demonstrated the impact of aMAP stratification on the prognosis of different SLD subclassifications using Kaplan–Meier survival curves. Figures 6A–C demonstrate the association between aMAP score stratification and survival outcomes within each SLD subclassification. The aMAP score effectively stratified participants into low-risk (aMAP < 50), medium-risk (aMAP 50–60), and high-risk (aMAP > 60) categories, successfully differentiating mortality risk across all three SLD subclassifications.

### 3.3 External validation of the association between aMAP score and MASLD in an independent hospital-based cohort

**Figure 7:**
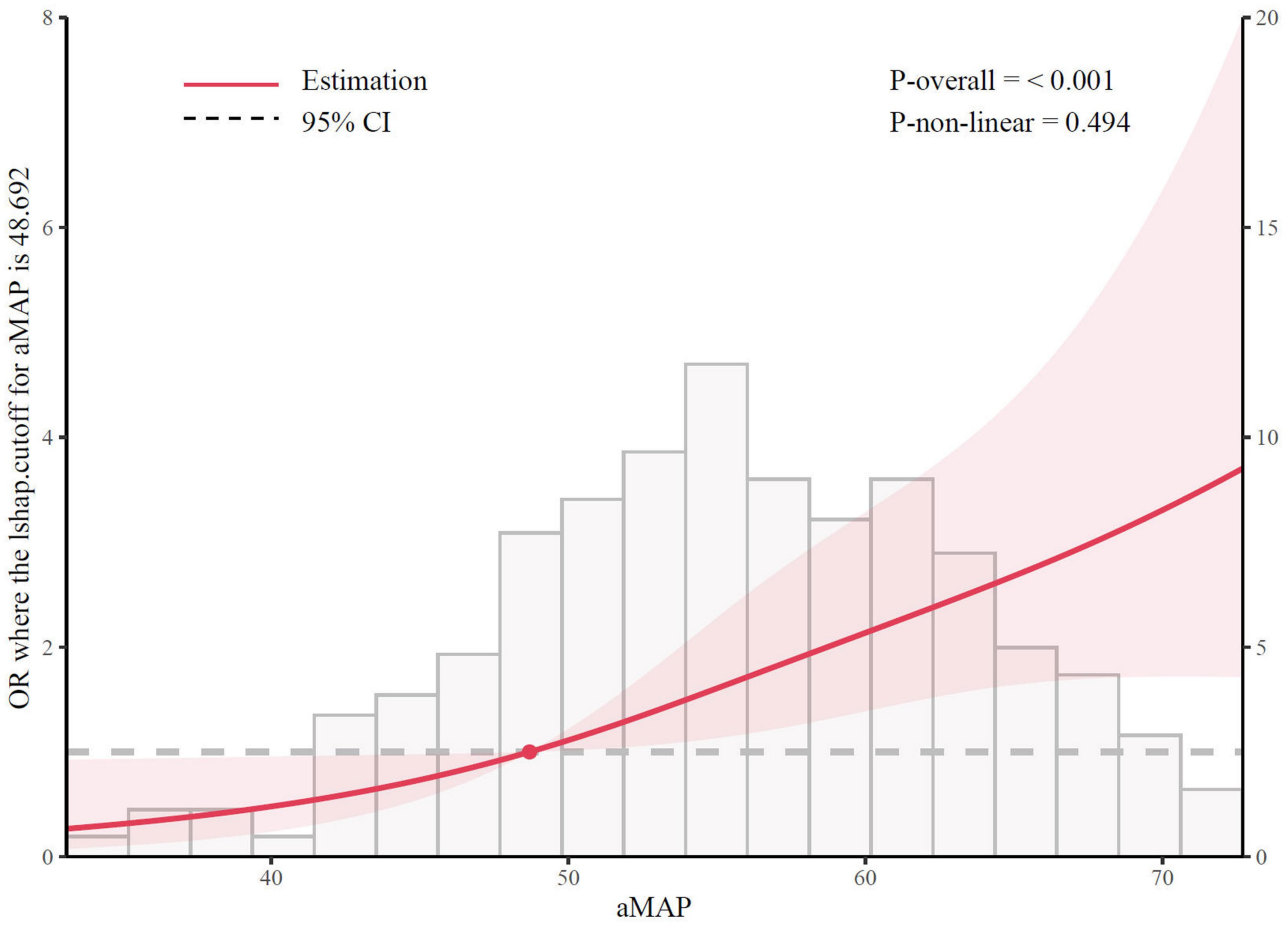
The association between aMAP and MASLD. aMAP, the age–male–ALBI–platelets; MASLD: metabolic dysfunction-associated steatotic liver disease; OR: odds ratio.

In the independent hospital-based cohort, a total of 642 participants were enrolled, including 96 patients with MASLD. Baseline characteristics were collected and aMAP scores were calculated for all participants to further validate the association between the aMAP score and MASLD status. Higher aMAP scores were consistently associated with the presence of MASLD (Table 5). As a continuous variable, elevated aMAP remained significantly associated with MASLD (OR 1.065, 95% CI 1.035–1.095; *p* < 0.01). When categorized into 3 groups, aMAP demonstrated a clear dose–response relationship with MASLD risk. Compared with the low-risk group, medium-risk group and high-risk group showed progressively increased odds of MASLD, with odds ratios of 2.229 (95% CI 1.150–4.676) and 3.538 (95% CI 1.816–7.456), respectively. RCS analysis indicated a significant positive association between aMAP and MASLD (P for overall < 0.01), without evidence of nonlinearity (P for nonlinearity = 0.494) (Figure 7). These findings provide external support for the robustness of the association between aMAP score and MASLD observed in the primary analysis.

**Table 5:** Association of aMAP with MASLD in external validation cohort.

aMAP	OR (95%CI)	*P-*value
Continuous	1.065(1.035, 1.095)	<0.01
Groups		
≤50	Reference	–
50–60	2.229(1.150, 4.676)	<0.01
>60	3.538(1.816, 7.456)	<0.01
*P* for trend		<0.01
AUC (95%CI)	0.65(0.593, 0.706)	

aMAP, the age–male–ALBI–platelets; MASLD: Metabolic dysfunction-associated steatotic liver disease; OR: odds ratio.

## 4 Discussion

In this prospective cohort study based on NHANES 1999–2018, we evaluated the association between aMAP score and mortality across different SLD subclassifications. Among 32,654 participants, we identified hepatic steatosis in 45.17% of individuals, the prevalence of MASLD, MetALD, and ALD as 41.14, 2.22 and 0.79%, respectively. This prevalence is consistent with other cycles of NHANES studies and other large-scale cohort investigations (15–17). Notably, 92% of ALD patients exhibited at least one cardiometabolic risk factor, suggesting that metabolic factors play a crucial role in liver disease progression even among heavy drinkers. After multivariable adjustment, compared to individuals without SLD, patients with MASLD, MetALD, and ALD demonstrated 11, 39, and 87% increased risks of all-cause mortality, respectively. This progressive increase in mortality risk across SLD subclassifications has been observed in previous studies (18). Our findings further highlight the complex associations of cardiometabolic risk factors and alcohol consumption with mortality across SLD subclassifications (19, 20). Beyond SLD classification, age, male sex, smoking, socioeconomic factors, and the aMAP score were independently associated with mortality (21). In all SLD subclassifications, RCS analysis revealed a non-linear relationship between aMAP and all-cause mortality, and Kaplan–Meier curves demonstrated that higher aMAP risk stratification was associated with increased mortality rates.

The aMAP score was initially developed for HCC risk stratification across various types of chronic liver disease and has demonstrated robust external validation in other studies (22). However, further validation in SLD populations remains necessary. Recently, a study revealed that MASLD represents a significant determinant of HCC risk following sustained virologic response in Japanese patients, with aMAP serving as an independent risk factor (23). In NHANES 1999–2018, the mean aMAP scores were 43, 47, 48, and 48 for participants without SLD, with MASLD, MetALD, and ALD, respectively. More than 10% of SLD patients were classified as high-risk (aMAP >60), a threshold that has been associated in other studies with annual HCC incidence rates of 1.6–4% (7). In contrast, FIB-4 demonstrated a different pattern. A multicenter study recommended enhanced HCC surveillance for MASLD patients with FIB-4 ≥ 3.25 (24). In our analysis, the proportion of individuals with FIB-4 > 2.67 increased progressively with alcohol consumption, showing a higher prevalence in ALD compared to MetALD, which in turn exceeded MASLD. This divergence likely reflects the distinct focus of these scoring systems: FIB-4 primarily evaluates hepatic fibrosis, while aMAP provides broader population-based risk stratification. Consistent with a retrospective cohort study from the national Veterans Health Administration, our finding underscores the urgency of implementing risk stratification strategies in SLD populations, particularly closely monitoring high-risk individuals for early detection of serious complications, such as cirrhosis and HCC (25).

Importantly, we complemented the population-based NHANES analysis with an independent hospital-based cohort from Southern China. In this external validation cohort, higher aMAP scores were consistently associated with increased odds of MASLD, and spline analyses suggested an overall approximately linear relationship. Despite differences in study design, population characteristics, and diagnostic approaches, these findings support the robustness and generalizability of the aMAP–MASLD association across distinct clinical settings.

The aMAP score was developed based on the ALBI (albumin-bilirubin) score (12), integrating age, sex, albumin, total bilirubin, and platelets, thereby reflecting the degree of liver fibrosis and prognosis to some extent. Because age is a component of aMAP and a major determinant of all-cause mortality, part of the prognostic signal of aMAP for this endpoint may reflect age. Nevertheless, albumin, bilirubin, platelet count, and sex may still add clinically relevant information on liver function and fibrosis burden. Studies have validated the excellent performance of aMAP alone or in combination with liver stiffness measurement (LSM) for predicting liver fibrosis in both hepatitis and fatty liver disease populations (8, 26). Additionally, aMAP has shown utility in assessing prognosis in breast cancer patients with liver metastases (27). A previous NHANES III study demonstrated that FIB-4 had limited ability to stratify mortality risk across different causes of death in SLD participants (15). Moreover, the prognostic impact of concurrent SLD in patients undergoing different HCC treatments, such as radiofrequency ablation, liver resection and orthotopic liver transplantation, remains inconsistent across other studies (28, 29). Therefore, we further analyzed the impact of aMAP on prognosis across SLD subclassifications. Our results showed that the three-tier aMAP risk stratification was associated with differences in long-term mortality risk across SLD subclassifications, with competing risk analysis confirming that the high aMAP group exhibited significantly elevated risks for all-cause mortality, cardiovascular mortality and cancer mortality. Although novel biomarkers such as GALAD (30) and PAaM (31) have demonstrated superior diagnostic and prognostic performance compared to aMAP in some cohorts, they typically require more complex laboratory assessments including tumor markers. In contrast, aMAP requires only two routine laboratory tests, making it particularly suitable for implementation in primary healthcare settings and pre-operative risk assessment, and potentially contributing to the challenges of surveillance for reducing liver cancer burden (32).

Our study has several notable strengths. First, this NHANES-based investigation included a large sample size with up to 20 years of follow-up, which strengthens the robustness of the findings and supports their generalizability within the US population. Second, we employed the most recent SLD classification criteria to systematically evaluate prognostic differences among MASLD, MetALD, and ALD, while utilizing aMAP for further risk stratification. This approach revealed differences in specific mortality types and provided a practical and accessible risk stratification tool that facilitates individualized management in clinical practice.

Several limitations should be acknowledged in this study. First, hepatic steatosis diagnosis was based on the FLI, a noninvasive score, rather than the gold standards of ultrasound, elastography, MRI, or liver biopsy. Second, alcohol consumption was based on self-reporting rather than objective biomarkers, which may lead to underestimation by both participants and researchers, particularly in MetALD and ALD populations (33, 34). Third, because liver-related mortality data are restricted from public use, we were unable to analyze liver-specific outcomes (such as cirrhosis and hepatic failure), which may have resulted in an underestimation of the prognostic value of the aMAP score. Fourth, although the maximum follow-up reached 20 years, participants enrolled in the more recent NHANES cycles had shorter follow-up durations, and the 20-year analyses were driven mainly by earlier cycles and should be interpreted with caution. Finally, although aMAP has been validated in multiple international cohorts, this study was based solely on the US population. Therefore, its applicability across different ethnic and geographic populations requires further evaluation in well-designed multicenter or single-center prospective study.

## 5 Conclusion

In conclusion, this study demonstrates that the aMAP score is independently associated with long-term mortality across different steatotic liver disease subclassifications in a large population-based cohort. By enabling effective risk stratification, aMAP may help identify individuals at increased risk and support more targeted clinical surveillance and resource allocation. These findings, further supported by an independent hospital-based validation cohort, underscore the potential value of aMAP as a pragmatic tool in the management of steatotic liver disease. Future studies are warranted to confirm its performance in diverse populations and to define its role within integrated risk assessment strategies.

## Data Availability

Publicly available datasets were analyzed in this study. This data can be found here: the NHANES datasets analyzed in this study are publicly available from the National Center for Health Statistics at https://wwwn.cdc.gov/nchs/nhanes/. The hospital-based validation cohort contains clinical data that are not publicly available due to privacy and ethical restrictions but can be accessed from the corresponding authors upon reasonable request. The datasets supporting the conclusions of this study are available in the NHANES database (https://wwwn.cdc.gov/nchs/nhanes/Default.aspx).

## Footnotes

1
https://www.cdc.gov/nchs/linked-data/mortality-files/
