# Supplementary material for: Association between the aMAP risk score and mortality in the MASLD/MetALD/ALD patient population: a cohort study
Source: Front Med (Lausanne). 2026 Apr 24;13:1799986. doi: 10.3389/fmed.2026.1799986 (PMC13154603; doi:10.3389/fmed.2026.1799986)
Supplement: Supplementary file 2 [file Table_1.DOCX]

$$\boldsymbol{FLI}=\frac{e^{0.953\ln\left( TG \right)+0.139BMI+0.718\ln\left( GGT \right)+0.053WC-15.745}}{1+e^{0.953\ln\left( TG \right)+0.139BMI+0.718\ln\left( GGT \right)+0.053WC-15.745}}\times100$$

$$\boldsymbol{US FLI}=\frac{e^{-0.8073\times non-Hispanicblack+0.3458\times MexicanAmerican+0.0093\times age+0.6151\times\ln\left( GGT \right)+0.0249\times WC+1.1792\times\ln\left( insulin \right)+0.8242\times\ln\left( glucose \right)-14.7812}}{1+e^{-0.8073\times non-Hispanicblack+0.3458\times MexicanAmerican+0.0093\times age+0.6151\times\ln\left( GGT \right)+0.0249\times WC+1.1792\times\ln\left( insulin \right)+0.8242\times\ln\left( glucose \right)-14.7812}}\times100.$$

$$\boldsymbol{ALBI score}=(log₁₀bilirubin[\mu mol/L]\times0.66)+(albumin[g/L]\times-0.085)$$

$$\boldsymbol{aMAP risk score}=\frac{(((0.06\times age[year]+0.89\times sex(Male:1,Female:0)+0.48\times[(log₁₀bilirubin[\mu mol/L]\times0.66)+(albumin[g/L]\times-0.085)]-0.01\times platelets[{10}^{9}/L])+7.4)))\times100}{14.77}$$

$$\boldsymbol{FIB-4 score}=\frac{Age[year]\times AST[U/L]}{platelets[{10}^{9}/L])\times{(ALT[U/L])}^{1/2}}$$

$$\boldsymbol{NFS}=-1.675+0.037\times age[year]+0.094\times BMI[kg/m^{2}]+1.13\times diabetes(yes=1,no=0)+(0.99\times AST/ALT radio)+(0.013\times platelets[{10}^{9}/L])-(0.66\times albumin[g/dL])$$

$$\boldsymbol{MAF-5}=-11.3674+WC[cm]\times0.0282-BMI[kg/m^{2}]\times0.1761+WC[cm]\times BMI[kg/m^{2}]\times0.0019+2.0762for diabetes+ln(AST[U/L])\times2.9207-0.0059\times platelets[{10}^{9}/L]$$

Formulas for different indicators. Note: FLI: fatty liver index; US FLI: U.S. fatty liver index; ALBI score: albumin-bilirubin score; aMAP risk score: age–male–ALBI–platelets risk score; FIB-4 score: fibrosis-4; NFS: NAFLD fibrosis score; MAF-5: metabolic dysfunction–associated fibrosis-5. TG: triglycerides; BMI: body mass index; WC: waist circumference; GGT: gamma-glutamyl transferase; ALT: alanine aminotransferase; AST: aspartate aminotransferase.
