# Supplementary material for: Association between the aMAP risk score and mortality in the MASLD/MetALD/ALD patient population: a cohort study
Source: Front Med (Lausanne). 2026 Apr 24;13:1799986. doi: 10.3389/fmed.2026.1799986 (PMC13154603; doi:10.3389/fmed.2026.1799986)
Supplement: Supplementary file 3 [file Table_2.DOCX]

| ***Baseline characteristics of NHANES 1999-2018 participants with weights*** | | | | | |
| --- | --- | --- | --- | --- | --- |
|  | **No SLD**  N = 82,744,899 | **MASLD**  N = 57,396,139 | **MetALD**  N = 3,785,982 | **ALD**  N = 1,129,784 | **P value** |
| Age, Mean (SD), years | 43.4 (16.9) | 48.7 (15.8) | 46.5 (14.3) | 44.9 (13.2) | <0.001 |
| Sex, n (%) |  |  |  |  | <0.001 |
| Male | 35,265,234 (42.6%) | 31,942,589 (55.7%) | 2,691,724 (71.1%) | 997,758 (88.3%) |  |
| Female | 47,479,666 (57.4%) | 25,453,549 (44.3%) | 1,094,259 (28.9%) | 132,026 (11.7%) |  |
| Race/ethnicity |  |  |  |  | <0.001 |
| Mexican American | 5,527,794 (6.7%) | 5,407,942 (9.4%) | 323,158 (8.5%) | 133,124 (11.8%) |  |
| Other Hispanic | 10,106,541 (12.2%) | 5,785,053 (10.1%) | 233,660 (6.2%) | 104,860 (9.3%) |  |
| Non-Hispanic White | 59,749,784 (72.2%) | 41,094,384 (71.6%) | 2,989,366 (79.0%) | 780,307 (69.1%) |  |
| Non-Hispanic Black | 7,360,781 (8.9%) | 5,108,760 (8.9%) | 239,799 (6.3%) | 111,492 (9.9%) |  |
| Marital status, n (%) |  |  |  |  | <0.001 |
| Married | 45,047,882 (54.4%) | 35,693,211 (62.2%) | 1,956,452 (51.7%) | 362,571 (32.1%) |  |
| Widowed/Divorced/Separated | 13,117,146 (15.9%) | 10,664,507 (18.6%) | 791,904 (20.9%) | 336,250 (29.8%) |  |
| Never married | 24,579,871 (29.7%) | 11,038,420 (19.2%) | 1,037,627 (27.4%) | 430,962 (38.1%) |  |
| Education level, n (%) |  |  |  |  | <0.001 |
| Less than high school | 11,548,217 (14.0%) | 9,606,650 (16.7%) | 538,026 (14.2%) | 289,352 (25.6%) |  |
| High school | 17,883,084 (21.6%) | 14,640,241 (25.5%) | 1,097,468 (29.0%) | 406,195 (36.0%) |  |
| Above high school | 53,313,598 (64.4%) | 33,149,248 (57.8%) | 2,150,488 (56.8%) | 434,237 (38.4%) |  |
| PIR, Mean (SD) | 3.1 (1.6) | 3.0 (1.6) | 3.2 (1.6) | 2.7 (1.6) | <0.001 |
| Smoking, n (%) | 46,789,868 (56.5%) | 30,417,543 (53.0%) | 1,049,425 (27.7%) | 186,905 (16.5%) | <0.001 |
| Drinking, Mean (SD), g/week |  |  |  |  |  |
| Male | 77.1 (136.0) | 37.0 (52.3) | 281.0 (55.9) | 671.0 (383.8) | <0.001 |
| Female | 31.5 (67.7) | 11.1 (23.2) | 200.4 (56.5) | 578.0 (343.6) | <0.001 |
| Number of CMRFs |  |  |  |  | <0.001 |
| 0 | 16,952,446 (20.5%) | 0 (0.0%) | 0 (0.0%) | 22,253 (2.0%) |  |
| 1 | 28,940,247 (35.0%) | 6,599,171 (11.5%) | 510,152 (13.5%) | 130,708 (11.6%) |  |
| 2 | 20,640,685 (24.9%) | 15,520,036 (27.0%) | 1,376,534 (36.4%) | 356,864 (31.6%) |  |
| 3 | 8,961,623 (10.8%) | 14,427,408 (25.1%) | 816,250 (21.6%) | 345,664 (30.6%) |  |
| 4 | 4,695,452 (5.7%) | 10,784,157 (18.8%) | 645,231 (17.0%) | 168,299 (14.9%) |  |
| 5 | 2,554,446 (3.1%) | 10,065,366 (17.5%) | 437,815 (11.6%) | 105,995 (9.4%) |  |
| Hypertension | 28,999,009 (35.0%) | 36,495,065 (63.6%) | 2,656,972 (70.2%) | 808,123 (71.5%) | <0.001 |
| Diabetes Mellitus | 5,177,822 (6.3%) | 14,885,993 (25.9%) | 543,967 (14.4%) | 131,414 (11.6%) | <0.001 |
| SLD: steatotic liver disease; MASLD: Metabolic dysfunction-associated steatotic liver disease; MetALD: metabolic and alcohol-related liver disease; ALD: alcohol-related liver disease; SD: standard deviation; CMRFs: cardiometabolic risk factors. Data presented as mean (SD) or n (%). Kruskal-Wallis test or Wilcoxon rank-sum test for continuous variables, and chi-square test or Fisher’s exact test for categorical variables. All estimates account for the NHANES complex survey design. | | | | | |
