# Supplementary material for: Association between the aMAP risk score and mortality in the MASLD/MetALD/ALD patient population: a cohort study
Source: Front Med (Lausanne). 2026 Apr 24;13:1799986. doi: 10.3389/fmed.2026.1799986 (PMC13154603; doi:10.3389/fmed.2026.1799986)
Supplement: Supplementary file 4 [file Table_3.DOCX]

| ***Laboratory characteristics of NHANES 1999-2018 participants with weights.*** | | | | | |
| --- | --- | --- | --- | --- | --- |
|  | **No SLD**  Weighted N = 82,744,899 | **MASLD**  Weighted N = 57,396,139 | **MetALD**  Weighted N = 3,785,982 | **ALD**  Weighted N = 1,129,784 | **P value** |
| AST, U/L | 22.0 (19.0, 26.0) | 23.0 (19.0, 28.0) | 26.0 (22.0, 33.0) | 30.0 (25.0, 41.0) | <0.001 |
| ALT, U/L | 19.0 (15.0, 24.0) | 25.0 (19.0, 34.0) | 29.0 (22.0, 42.0) | 35.0 (24.0, 50.0) | <0.001 |
| Platelets, 10⁹/L | 243.0 (209.0, 286.0) | 250.0 (212.0, 296.0) | 240.0 (204.0, 284.0) | 237.0 (202.0, 271.0) | <0.001 |
| Triglycerides, mg/dL | 89.0 (65.0, 124.0) | 165.0 (116.0, 242.0) | 173.0 (124.0, 262.0) | 180.0 (131.0, 273.0) | <0.001 |
| Total Bilirubin, μmol/L | 12.0 (8.6, 13.7) | 10.3 (8.6, 13.7) | 12.0 (8.6, 15.4) | 12.0 (8.6, 15.4) | <0.001 |
| Albumin, g/L | 44.0 (41.0, 46.0) | 42.0 (40.0, 44.0) | 44.0 (41.0, 46.0) | 43.0 (41.0, 46.0) | <0.001 |
| aMAP score | 42.7 (37.1, 49.5) | 46.3 (39.8, 52.9) | 47.0 (41.2, 52.6) | 47.1 (42.3, 53.7) | <0.001 |
| aMAP group, n (%) |  |  |  |  | <0.001 |
| < 50 | 63,318,328 (76.5%) | 37,101,623 (64.6%) | 2,447,700 (64.7%) | 678,431 (60.0%) |  |
| 50-60 | 15,080,369 (18.2%) | 15,810,274 (27.5%) | 1,087,623 (28.7%) | 373,868 (33.1%) |  |
| ≥ 60 | 4,346,202 (5.3%) | 4,484,242 (7.8%) | 250,659 (6.6%) | 77,485 (6.9%) |  |
| FIB-4 score | 1.0 ± 0.8 | 1.0 ± 0.8 | 1.1 ± 1.0 | 1.3 ± 1.0 | <0.001 |
| FIB-4 group, n (%) |  |  |  |  | <0.001 |
| < 1.3 | 64,954,655 (78.5%) | 43,578,798 (75.9%) | 2,736,252 (72.3%) | 759,567 (67.2%) |  |
| 1.3-2.67 | 16,119,012 (19.5%) | 12,560,921 (21.9%) | 896,991 (23.7%) | 274,288 (24.3%) |  |
| > 2.67 | 1,671,231 (2.0%) | 1,256,420 (2.2%) | 152,740 (4.0%) | 95,929 (8.5%) |  |
| MAF-5 score | -1.4 ± 1.3 | 1.5 ± 2.2 | 1.3 ± 2.1 | 1.7 ± 2.1 | <0.001 |
| NFS score | -2.7 (-3.5, -1.8) | -1.6 (-2.6, -0.5) | -2.0 (-2.9, -1.1) | -2.1 (-3.0, -1.1) | <0.001 |
| ALBI score | -3.0 (-3.2, -2.8) | -2.9 (-3.1, -2.7) | -3.0 (-3.2, -2.8) | -3.0 (-3.2, -2.8) | <0.001 |
| SLD: steatotic liver disease; MASLD: Metabolic dysfunction-associated steatotic liver disease; MetALD: metabolic and alcohol-related liver disease; ALD: alcohol-related liver disease; ALT: alanine aminotransferase; AST: aspartate transaminase; ALBI score: albumin-bilirubin score; aMAP: age–male–ALBI–platelets; FIB-4: Fibrosis-4; MAF-5: metabolic dysfunction–associated fibrosis-5; NFS: NAFLD fibrosis score. Data presented as median (Q1, Q3) or n (%). Kruskal-Wallis test or Wilcoxon rank-sum test for continuous variables, and chi-square test or Fisher’s exact test for categorical variables. All estimates account for the NHANES complex survey design. | | | | | |
