# Supplementary material for: Association between the aMAP risk score and mortality in the MASLD/MetALD/ALD patient population: a cohort study
Source: Front Med (Lausanne). 2026 Apr 24;13:1799986. doi: 10.3389/fmed.2026.1799986 (PMC13154603; doi:10.3389/fmed.2026.1799986)
Supplement: Supplementary file 5 [file Table_4.DOCX]

**Proportional Hazards Assumption Testing for Cox Models**

| **Mortality** | **All-cause Mortality** | | **CVD Mortality** | | **Cancer Mortality** | |
| --- | --- | --- | --- | --- | --- | --- |
| **Variable** | **Chi-square** | **p-value** | **Chi-square** | **p-value** | **Chi-square** | **p-value** |
| aMAP group | 5.492 | 0.064 | 0.174 | 0.917 | 1.480 | 0.477 |
| Sex | 5.675 | **0.017** | 1.094 | 0.296 | 0.556 | 0.456 |
| Race/ethnicity | 1.459 | 0.692 | 6.872 | 0.076 | 8.303 | **0.040** |
| Education level | 6.400 | **0.041** | 1.334 | 0.513 | 8.090 | **0.018** |
| Marital status | 8.840 | **0.012** | 5.055 | 0.080 | 6.085 | **0.048** |
| Smoking status | 2.252 | 0.133 | 0.667 | 0.414 | 0.387 | 0.534 |
| Poverty income ratio | 3.892 | 0.143 | 3.077 | 0.215 | 0.664 | 0.717 |
| Proportional hazards assumption testing was performed using Schoenfeld residuals based on conventional Cox models fitted in the same analytic sample as the primary weighted Cox models. A P value < 0.05 suggests possible violation of the proportional hazards assumption. aMAP: the age–male–ALBI–platelets. | | | | | | |
