# Supplementary material for: Association between the aMAP risk score and mortality in the MASLD/MetALD/ALD patient population: a cohort study
Source: Front Med (Lausanne). 2026 Apr 24;13:1799986. doi: 10.3389/fmed.2026.1799986 (PMC13154603; doi:10.3389/fmed.2026.1799986)
Supplement: Supplementary file 6 [file Table_5.DOCX]

**2-year landmark analysis among SLD population**

| **aMAP Group** | **HR (95%CI)** | **P value** | **Outcome** |
| --- | --- | --- | --- |
| <50 | 1.00 (Reference) | - | All-Cause Mortality |
| 50-60 | 4.71 (3.96-5.60) | <0.001 | All-Cause Mortality |
| >60 | 13.85 (11.56-16.60) | <0.001 | All-Cause Mortality |
| Trend test | - | <0.001 | All-Cause Mortality |
| <50 | 1.00 (Reference) | - | Cardiovascular Mortality |
| 50-60 | 5.34 (3.94-7.22) | <0.001 | Cardiovascular Mortality |
| >60 | 18.10 (13.68-23.94) | <0.001 | Cardiovascular Mortality |
| Trend test | - | <0.001 | Cardiovascular Mortality |
| <50 | 1.00 (Reference) | - | Cancer Mortality |
| 50-60 | 5.03 (3.69-6.87) | <0.001 | Cancer Mortality |
| >60 | 13.11 (9.29-18.50) | <0.001 | Cancer Mortality |
| Trend test | - | <0.001 | Cancer Mortality |

For the 2-year landmark analysis, participants who died or were censored within the first 2 years were excluded, and the fully adjusted Cox models were refitted in the remaining population. SLD: steatotic liver disease; HR: hazard ratio; CI, confidence interval; aMAP: the age–male–ALBI–platelets.
