# Supplementary material for: Association between the aMAP risk score and mortality in the MASLD/MetALD/ALD patient population: a cohort study
Source: Front Med (Lausanne). 2026 Apr 24;13:1799986. doi: 10.3389/fmed.2026.1799986 (PMC13154603; doi:10.3389/fmed.2026.1799986)
Supplement: Supplementary file 7 [file Table_6.DOCX]

*Characteristics and mortality comparison after imputation*

| **Category** | **No Imputation** | **After Imputation** | **P value** |
| --- | --- | --- | --- |
| **Total number, n** | 32654 | 36602 |  |
| **SLD classifications, n (%)** |  |  | **0.299** |
| No SLD | 17903 (55.4%) | 20338 (56.1%) |  |
| MASLD | 13434 (41.6%) | 14833 (40.9%) |  |
| MetALD | 724 (2.2%) | 788 (2.2%) |  |
| ALD | 259 (0.8%) | 285 (0.8%) |  |
| **Age groups, n (%)** |  |  | **0.319** |
| 20-39 years | 12396 (38.0%) | 13444 (37.6%) |  |
| 40-59 years | 10268 (31.4%) | 11164 (31.2%) |  |
| >=60 years | 9990 (30.6%) | 11121 (31.1%) |  |
| **Sex, n (%)** |  |  | **0.953** |
| Male | 15948 (48.8%) | 17868 (48.8%) |  |
| Female | 16706 (51.2%) | 18734 (51.2%) |  |
| **AMAP groups, n (%)** |  |  | **0.298** |
| <50 | 21421 (65.6%) | 24213 (66.2%) |  |
| 50-60 | 8018 (24.6%) | 8864 (24.2%) |  |
| >60 | 3215 (9.8%) | 3525 (9.6%) |  |
| **Mortality, n (%)** |  |  | **0.880** |
| All-cause mortality | 4572 (14.0%) | 5090 (13.9%) |  |
| CVD mortality | 1445 (4.4%) | 1584 (4.3%) |  |
| Cancer mortality | 1034 (3.2%) | 1138 (3.1%) |  |
| Other-cause mortality | 2093 (6.4%) | 2368 (6.5%) |  |

Note: P values are reported only for block-level comparisons. SLD classifications, age groups, sex distribution, AMAP groups, and mortality distribution were compared using overall categorical tests.
