# Supplementary material for: Association between the aMAP risk score and mortality in the MASLD/MetALD/ALD patient population: a cohort study
Source: Front Med (Lausanne). 2026 Apr 24;13:1799986. doi: 10.3389/fmed.2026.1799986 (PMC13154603; doi:10.3389/fmed.2026.1799986)
Supplement: Supplementary file 8 [file Table_7.DOCX]

Association between All-Cause Mortality with aMAP groups among the total population and the SLD population

|  |  | **Model 1** | | **Model 2** | | **Model 3** | |
| --- | --- | --- | --- | --- | --- | --- | --- |
| **Population** | **aMAP Group** | **HR (95%CI)** | ***P value*** | **HR (95%CI)** | ***P value*** | **HR (95%CI)** | ***P value*** |
| Total Population | <50 | 1.00 (Reference) | - | 1.00 (Reference) | - | 1.00 (Reference) | - |
|  | 50-60 | 6.02 (5.44-6.67) | <0.001 | 5.76 (5.20-6.39) | <0.001 | 5.38 (4.78-6.06) | <0.001 |
|  | >60 | 20.60 (18.50-22.93) | <0.001 | 19.00 (16.99-21.24) | <0.001 | 16.37 (14.45-18.54) | <0.001 |
|  | **Trend test** | **-** | **<0.001** | **-** | **<0.001** | **-** | **<0.001** |
| Total SLD Population | <50 | 1.00 (Reference) | - | 1.00 (Reference) | - | 1.00 (Reference) | - |
|  | 50-60 | 4.55 (3.95-5.23) | <0.001 | 4.37 (3.78-5.04) | <0.001 | 4.38 (3.71-5.18) | <0.001 |
|  | >60 | 15.03 (12.91-17.50) | <0.001 | 13.76 (11.78-16.07) | <0.001 | 12.61 (10.59-15.01) | <0.001 |
|  | **Trend test** | **-** | **<0.001** | **-** | **<0.001** | **-** | **<0.001** |
| aMAP, the age–male–ALBI–platelets; HR: hazard ratio; CI, confidence interval; SLD: steatotic liver disease. All models were survey-weighted and accounted for NHANES complex survey design. Model 1: Unadjusted; Model 2: adjusted race/ethnicity; Model 3: adjusted for race/ethnicity, poverty income ratio (PIR), education level, marital status and smoking. | | | | | | | |
