# Supplementary material for: Association between the aMAP risk score and mortality in the MASLD/MetALD/ALD patient population: a cohort study
Source: Front Med (Lausanne). 2026 Apr 24;13:1799986. doi: 10.3389/fmed.2026.1799986 (PMC13154603; doi:10.3389/fmed.2026.1799986)
Supplement: Supplementary file 9 [file Table_8.DOCX]

***Predictive performance of aMAP components for all-cause mortality***

| **Variables** | **5-year AUC** | **10-year AUC** | **20-year AUC** |
| --- | --- | --- | --- |
| Age | **0.819** | **0.837** | **0.872** |
| Albumin | 0.402 | 0.425 | 0.359 |
| Platelets | 0.393 | 0.365 | 0.393 |
| Sex | 0.551 | 0.546 | 0.552 |
| Total Bilirubin | 0.521 | 0.528 | 0.679 |
| **aMAP** | 0.788 | 0.809 | 0.833 |

aMAP: age-male-albumin-bilirubin-platelets; AUC: area under the curve.
