# Supplementary material for: Association between the aMAP risk score and mortality in the MASLD/MetALD/ALD patient population: a cohort study
Source: Front Med (Lausanne). 2026 Apr 24;13:1799986. doi: 10.3389/fmed.2026.1799986 (PMC13154603; doi:10.3389/fmed.2026.1799986)
Supplement: Supplementary file 10 [file Table_9.DOCX]

**Univariate time-dependent ROC analysis stratified by age cutoff**

| **Age group** | **Marker** | **5-year AUC** | **10-year AUC** | **20-year AUC** |
| --- | --- | --- | --- | --- |
| ≤60 | ALBI | 0.705 | 0.690 | 0.700 |
|  | FIB-4 | 0.726 | 0.728 | 0.693 |
|  | MAF-5 | 0.723 | 0.735 | 0.702 |
|  | NFS | 0.736 | 0.753 | 0.724 |
|  | aMAP | 0.745 | 0.759 | 0.744 |
| >60 | ALBI | 0.655 | 0.668 | 0.787 |
|  | FIB-4 | 0.671 | 0.692 | 0.717 |
|  | MAF-5 | 0.638 | 0.666 | 0.714 |
|  | NFS | 0.684 | 0.709 | 0.788 |
|  | aMAP | 0.701 | 0.720 | 0.771 |

Abbreviations: ALBI, albumin-bilirubin; FIB-4, fibrosis-4 index; NFS, NAFLD fibrosis score; aMAP, age-male-ALBI-platelets.
