# Supplementary material for: Association between the aMAP risk score and mortality in the MASLD/MetALD/ALD patient population: a cohort study
Source: Front Med (Lausanne). 2026 Apr 24;13:1799986. doi: 10.3389/fmed.2026.1799986 (PMC13154603; doi:10.3389/fmed.2026.1799986)
Supplement: Supplementary file 11 [file Table_10.DOCX]

Cumulative incidences of cause-specific death for the SLD population by aMAP groups

| Characteristic | N | N Event | Years 10 | Years 20 | p value¹ |
| --- | --- | --- | --- | --- | --- |
| **Cardiovascular mortality** | 14417 | 755 |  |  | <0.001 |
| Low (<50) |  | 159 | 1.4% (1.1%, 1.6%) | 4.8% (6.0%, 6.0%) |  |
| Medium (50-60) |  | 315 | 7.0% (6.0%, 7.9%) | 17.9% (20.8%, 20.8%) |  |
| High (>60) |  | 281 | 17.3% (15.1%, 19.5%) | 33.6% (37.7%, 37.7%) |  |
| **Cancer mortality** | 14417 | 505 |  |  | <0.001 |
| Low (<50) |  | 132 | 1.2% (0.9%, 1.5%) | 4.1% (5.2%, 5.2%) |  |
| Medium (50-60) |  | 214 | 4.9% (4.2%, 5.7%) | 10.9% (13.0%, 13.0%) |  |
| High (>60) |  | 159 | 11.8% (9.9%, 13.7%) | 16.2% (19.0%, 19.0%) |  |
| Levels of significance as shown in the table (Fine-Grey’s test). SLD: steatotic liver disease; aMAP, the age–male–ALBI–platelets. | | | | | |
