# Supplementary material for: Association between the aMAP risk score and mortality in the MASLD/MetALD/ALD patient population: a cohort study
Source: Front Med (Lausanne). 2026 Apr 24;13:1799986. doi: 10.3389/fmed.2026.1799986 (PMC13154603; doi:10.3389/fmed.2026.1799986)
Supplement: Supplementary file 12 [file Table_11.DOCX]

Cumulative incidences of cause-specific death among SLD subclassification population

| Characteristic | N | N Event | Years 10 | Years 20 | p value |
| --- | --- | --- | --- | --- | --- |
| **Cardiovascular mortality** | 14417 | 755 |  |  | 0.663 |
| MASLD |  | 709 | 4.7% (4.3%, 5.1%) | 11.3% (10.1%, 12.5%) |  |
| MetALD |  | 33 | 4.1% (2.4%, 5.8%) | 9.5% (5.8%, 13.3%) |  |
| ALD |  | 13 | 4.5% (1.6%, 7.4%) | 8.3% (3.6%, 13.0%) |  |
| **Cancer mortality** | 14417 | 505 |  |  | 0.627 |
| MASLD |  | 465 | 3.3% (3.0%, 3.7%) | 7.2% (6.2%, 8.1%) |  |
| MetALD |  | 29 | 3.8% (2.2%, 5.4%) | 7.3% (4.3%, 10.4%) |  |
| ALD |  | 11 | 4.1% (1.3%, 7.0%) | 9.7% (2.3%, 17.1%) |  |
| Levels of significance as shown in the table (Fine-Grey’s test). SLD: steatotic liver disease; MASLD: Metabolic dysfunction-associated steatotic liver disease; MetALD: metabolic and alcohol-related liver disease; ALD: alcohol-related liver disease. | | | | | |
